# Rediscover and Refine QTLs for Pig Scrotal Hernia by Increasing a Specially Designed F_3_ Population and Using Whole-Genome Sequence Imputation Technology

**DOI:** 10.3389/fgene.2019.00890

**Published:** 2019-09-23

**Authors:** Wenwu Xu, Dong Chen, Guorong Yan, Shijun Xiao, Tao Huang, Zhiyan Zhang, Lusheng Huang

**Affiliations:** State Key Laboratory for Pig Genetic Improvement and Production Technology, Jiangxi Agricultural University, Nanchang, China

**Keywords:** GWAS, imputation, haplotype, specially designed population, scrotal hernia, pigs

## Abstract

Pig scrotal hernia is one of the most common congenital defects triggered by both genetic and environmental factors, leading to severe economic loss as well as poor animal welfare in the pig industry. Identification and implementation of genomic regions controlling scrotal hernia in breeding is of great appeal to reduce incidences of hernia in pig production. The aim of this study was to identify such regions or molecular markers affecting scrotal hernia in pigs. First of all, we summarized and analyzed the results of some international teams on scrotal hernia and designed a specially population which contains 246 male individuals. We then performed genome-wide association study (GWAS) in this specially designed population using two scenarios, i.e., the target panel data before and after imputation, which contain 42,365 SNPs and 18,756,672 SNPs, respectively. In addition, a series of methods including genetic differentiation analysis, linkage disequilibrium and linkage analysis (LDLA), and haplotype sharing analysis were appropriate to provide for further analysis to identify the potential gene underlying the QTL. The GWAS in this report detected a highly significant region affecting scrotal hernia within a 24.8Mb region (114.1–138.9Mb) on SSC8. And the result of genetic differentiation analysis also showed a strong genetic differentiation signal between 116.1 and 132.7Mb on SSC8. In addition, the QTL interval was refined to 2.99Mb by combining LDLA and genetic differentiation analysis. Finally, two susceptibility haplotypes were identified through haplotype sharing analysis, with one potential causal gene in it. Our study provided deeper insights into the genetic architecture of pig scrotal hernia and contributed to further fine-mapping and characterize haplotype and gene that influence scrotal hernia in pigs.

## Introduction

Pig hernias are of the most common congenital defects which cause severe economic losses as well as poor animal welfare in the pig industry. The most common types of hernias in pig are scrotal and umbilical hernia. Scrotal hernia is the phenomenon of abdominal contents falling into scrotum from the unilateral or bilateral inguinal rupture, causing local expansion bulge (Grindflek et al., 2006; Du et al., 2009; Zhao et al., 2009). As a complex congenital defect, the reason of scrotal hernia formation is unclear; some abnormal phenomena and problems occurred at the stage of the development and obliteration of processus vaginalis in descent of testis, which have been considered to be the main reason for the development of scrotal hernia (Clarnette and Hutson, 1997; Clarnette et al., 1998). The genetic mechanical of scrotal hernia is also poorly clarified, only with the knowledge of cause by both multiple genetic and environmental factors. In the pig breeding industry, the occurrence of scrotal hernia is varied from 1.7 to 6.7% across from pig breeds and populations, and the heritability estimation varied from 0.2 to 0.6 in disparate studies (Mikami and Fredeen, 1979; Thaller et al., 1996). Environmental factors, as a potential factor in the occurrence of complex genetic diseases, have a great influence on the occurrence of scrotal hernia. Research reports showed that the incidence of scrotal hernia in Dutch Landrace and large white pig was 1.36 and 1.31%, respectively, while the corresponding incidence rate of Dutch Landrace and large white pig of Hypor was 0.54 and 0.22% (PK, 2006). In 2010, the European Breeding Corporation reported that the incidence of scrotal hernia in Dutch Landrace and large white pig was 0.383% (Walters, 2010). Obviously, the difference of environment will make the incidence of scrotal hernia different.

In breeding practice, it is not effective to decrease the incidence of pig scrotal hernia by conventional phenotypic selection. One of the methods of hernia resistance breeding is to isolate and identify susceptibility loci and major causative genes and then implement marker assisted selection. Currently, several research groups have identified the susceptible loci and potential positional candidate genes for scrotal hernia. Grindflek et al. reported several susceptibility QTLs for pig scrotal hernias on eight chromosomes (Grindflek et al., 2006). Ding et al. have revealed seven regions on SSC2, 4, 8, 10, 13, 16, and 18 for scrotal hernia in a White Duroc and Erhualian F_2_ intercross using nonparametric genome-wide linkage (NPL) analysis and transmission disequilibrium test (TDT) (Ding et al., 2009). Du et al. found that four regions surrounding *ELF5, KIF18A, COL23A1* on chromosome 2, and *NPTX1* on chromosome 12 may contain the genetic variants important for the development of the scrotal hernia development using a family-based analysis (Du et al., 2009). Sevillano et al. reported a susceptibility region on SSC13 between 34 and 37 Mb for scrotal hernia (Sevillano et al., 2015). However, these susceptibility areas are rarely further confirmed in other research groups; even using bigger population sizes, the genetic control of scrotal hernia has still not been clarified.

In the 10 years, we performed two statistical methods (TDT and NPL) in the F_2_ population using 194 microsatellites and identified one chromosomal region distributed on SSC8 for the scrotal hernia. Generally speaking, nonparametric linkage analysis (NPL) evaluates allele sharing among affected individuals and comes to a result without particular model assumptions, and the TDT was proposed as a family-based association test for the presence of genetic linkage between a genetic marker and a trait; more computational details with this 2 statistical methods were showed by Ding et al. (2009). Using the same population, we perform GWAS study in 60K genotypes, the result manifested that none of SNPs achieved the genome-wide significance threshold (Su et al., 2014). The feasible reasons for the “missing QTLs” in GWAS study probably are the low linkage disequilibrium between markers and low incidence rate in the subject population, or due to the intricacy genetic basis of this congenital defect. To overcoming these problems and exploring this congenital defect, we designed a specially F_3_ population which was mated with full-sibs or half-sib of the affected individuals and imputed the chip SNPs to whole-genome sequences (**Supplementary Figure S1**) then implemented several classical genetic methods to rediscover and refine QTLs for pig scrotal hernia. Our aim in this study was to identify susceptibility loci of pig scrotal hernia and provided a novel insight for further analysis of the genetic basis of this congenital defect.

## Material and Method

All procedures including experimental animals established and tissue collection were performed in accordance with the guidelines approved by the Ministry of Agriculture of China. This study was approved by the ethics committee of Jiangxi Agricultural University.

## Animals of the Target Population

A four-generation resource population was developed from the intercross of 2 White Duroc boars (PIC 1075) and 17 Chinese Erhualian sows between 2,000 to 2,006. In briefly, two White Duroc boars were crossed to 17 Erhualian sows, then 9 F_1_ boars, and 59 F_1_ sows were randomly selected to produce a total of 1,912 F_2_ pigs in 6 batches avoiding full-sib mating (Guo et al., 2009). Last, 62 F_2_ boars and 149 F_2_ sows were selected to produce two types of F_3_ population. The ordinary experiment population contains 661 F_3_ offspring from an intercross of randomly chosen F_2_ avoiding full-sib mating; the particular hernia population in this study contains 851 F_3_ offspring, which were designed to mate the health full-sibs or half-sibs of affected individuals. Affected pigs were diagnosed and recorded carefully by veterinarians at three age stages: 46, 90, and 240 days. In summary, 23 affected pigs from F_2_ population were confirmed, 5 affected pigs from ordinary F_3_ population, and 23 affected pigs from F_3_ hernia study population were diagnosed, respectively. A total of 1,020 individuals (19 F_0_, 68 F_1_, and 933 F_2_) and 500 F_3_ were genotyped. For this study, 246 male F_3_ pigs were chosen for GWAS analysis, which contain 18 available DNA samples for affected individuals. Furthermore, 19 F_0_, 68 F_1_, and 516 F_2_ male pigs, and 246 F_3_ male pigs were used in haplotype sharing analysis.

Genomic DNA was isolated from ear tissue with a standard phenol/chloroform extraction method. All DNA samples were qualified and diluted to a final concentration of 50 ng/µl in 96-well plates. A total of 1,020 F_2_ and 500 F_3_ were genotyped with the Illumina PorcineSNP60 BeadChip and GeneSeek GGP Porcine 50K BeadChip on an iScan System (Illumina, USA) following the manufacturer’s protocol, respectively (Ramos et al., 2009). Physical positions of SNPs on chromosomes referred to the swine reference genome sequence assembly (Sus_scrofa11.1) (http://asia.ensembl.org/Sus_scrofa/Info/Index). Quality control procedures were implemented by PLINK (version 1.07). Briefly, SNPs were removed if their positions on the genome build 11.1 were unspecific, call rate <90%, and minor allele frequency (MAF) <1%. Animals more than 10% missing genotypes were removed. To keep the alleles consistency with the sequencing data, we firstly aligned the primer sequences of each SNP to the reference porcine genome assembly Sus scrofa 11.1 by BLAST. Then, the genotypes of reversed SNP strands in target panel were flipped using PLINK (v1.9) software (Chang et al., 2015); SNPs without positions were excluded for further analysis.

## Haplotype Construction of Reference Panel

In this study, a wide collection of 109 whole-genome sequence individuals from 14 difference populations were used as a reference; each breed contained 2 to 22 individuals. More details on the origins, breeds, and sample size are shown in **Table 1**. We firstly trimmed the raw reads according to a quality score threshold greater than 15; then, BWA (Burrows–Wheeler Aligner) was used to align the raw reads which passed chastity filtering to the reference porcine genome assembly Sus scrofa11.1 (Li and Durbin, 2009). Variants were identified using the GATK (Genome Analysis Toolkit) (McKenna et al., 2010); PCR duplications were firstly marked by Picard MarkDuplicates (http://broadinstitute.github.io/picard/), and GATK IndelRealigner option was carried out for local realignments. Then, variants were filtered with GATK VariantFiltration option. VCFtools was used to remove the structural variants. Subsequently, the haplotypes of 109 individuals with cleaned SNP data were constructed by Beagle (v4.1) (Browning and Browning, 2007). Specifically, the number of markers to include in each sliding window was set to 100,000, and the overlap between windows was set to 3,000 markers. Then, the number of phasing iterations was set to 50. Finally, the other options involving in the imputation follow the default setting.

**Table 1:** The components of the reference panel.

Breeds	Sample Size	Depth	Location
Bamei	6	24.9	Shanxi, China
Hetao	6	24.4	Inner Mongolia, China
Laiwu	6	27.5	Shandong, China
Min	6	25.8	Heilongjiang, China
Bamaxiang	6	28.1	Guangxi, China
Luchuan	6	26.4	Guangxi, China
Wuzhishan	6	26.1	Hainan, China
Jinhua	6	26.2	Zhejiang, China
Erhualian	19	28.1	Jiangsu, China
Tibet	22	26.9	Southwest China
Baoshan	6	26.5	Yunnan, China
Neijiang	6	26.2	Sichuan, China
White Duroc	2	31.1	USA
Wild boar	6	28.9	South China; North China; Sumatra, Indonesia

## Imputation

Whole-genome sequence imputation between target and reference panel was conducted by Beagle (v4.1) using the default parameter settings (Browning and Browning, 2016). Specifically, the size of imputed region was set to 50,000 markers per window, and the overlap between windows was set to 3,000 SNPs. This software first constructed local haplotypes using the hidden Markov chain Monte Carlo (MCMC) algorithm and then resampled new estimated haplotypes for each individual based on a hidden Markov model (HMM).

Imputation accuracy should be further investigated in whole-genome sequence data because of the low density and common variants in 50k. Browning et al. and Williams et al. have fully exhibited the number of individuals present in a population is a crucial factor in determining how well the phase can be estimated for haplotype construction (Browning and Browning, 2011; Williams et al., 2012). Therefore, 109 whole-genome sequence pigs including 19 F_0_ who were the progenitor of the 500 F_3_ populations were also regarded as reference panel in order to obtain more accurate phase information. Then, the genotypic concordance rate and the squared correlation (R2) between best-guess imputed and the original variants as imputation accuracy. The genotypic concordance rate used a cross-validation strategy described in previous studies (Brondum et al., 2014; van Binsbergen et al., 2014; Pausch et al., 2017). More specifically, two thousand loci in the target sample were deleted randomly then imputed in the same strategy. The number of 2,000 alleles imputed correctly divided by total 2,000 loci (the allelic correct rate) was taken to calculate the accuracy of imputation. Finally, in order to balance the imputation accuracy and missing proportion in the next analysis process, we excluded the variants with call rate <90% and MAF <0.03.

## GWAS

GEMMA was utilized to perform the association analyses underlining the standard linear mixed model (Zhou and Stephens, 2012). Sex and batch were included as fixed effects. Heritability was estimated by using −lmm procedure implemented in GEMMA using genomic relationship matrix. Population stratification and were adjust by including genomic relationship matrix. Briefly, this model is denoted as:

$$y=W\alpha +X\beta +u+\in ;\;u\sim MVN_{n}(0,\lambda^{-1}K),\in \sim MVN_{n}(0,\lambda \tau^{-1}{\text{I}}_{n})$$

where y is a n element vector of phenotypic values (or case/control labels), α is a c-vector of fixed effects, *β* is the effect size of SNPs, W is a design matrix of covariates, x is a vector of genotypes at each locus, and u is the vector of random effects following the multivariate normal distribution MVN_n_(0, λτ^−1^K), where τ^−1^ is the variance of the residual errors, and λ is the ratio between τ^−1^ and the variance of the residual errors; K is a known kinship matrix, ∈ is an vector of errors following the multivariate distribution *MVN_n_*(0, λτ^−1^I*_n_*), and I*_n_* is an n × n identity matrix. Normally, significance threshold of multiple test in chip array-based GWAS was adjusted by naïve Bonferroni corrections, which is 0.05 divided number of examined SNPs. However, this approach would lead to over correction and decreasing the detection power in GWAS as these tests are non-independent for the linkage disequilibrium between markers. We herein used 5E−08 as a genome-wide suggestive significance threshold following Pe’er et al. and Johnson et al. (Pe’er et al., 2008; Johnson et al., 2010). The population stratification is one of the factors that affects the validity of genome-wide association study (Pearson and Manolio, 2008). To check if stratification exists in our result, quantile–quantile plots (Q–Q plots) were implemented to evaluate population stratification effects. The Q–Q plots were constructed with R software. Measures of linkage disequilibrium (r and r^2^) between SNPs were estimated by plink 1.07 (Clarnette and Hutson, 1997), the default settings for minimum linkage between SNPs at threshold r^2^ = 0.8.

## Genetic Differentiation Analysis

To elucidate whether there is genetic differentiation exist in scrotal hernia pigs and health pigs, we divided the affected pigs and the unaffected pigs into two groups, as the method did by Zhang et al. (2019) then assessed allele frequency differentiation using the unbiased genetic differentiation estimated of the fixation index (Fst). Akey et al. have fully described estimation of unbiased Fst fixation index in his paper using SNP dataset (Akey et al., 2002). Briefly, Fst was estimated as follows:

$$Fst=\frac{MSP-MSG}{MSP+(n_{c}-1)MSG}$$

where MSG represents the observed mean square errors for loci within populations, MSP denotes the observed mean square errors for loci between populations, and *n_c_* is the average sample size across samples, which incorporates and corrects for the variance in the sample size over population

$$MSG=\frac{1}{\sum_{i=1}^{s}n_{i}-1}\sum_{i}^{s}n_{i}p_{Ai}(1-p_{Ai})$$

$$MSP=\frac{1}{s-1}\sum_{i}^{s}n_{i}{\left(p_{Ai}-{\bar{p}}_{A}\right)}^{2}$$

$$n_{c}=\frac{1}{s-1}\sum_{i=1}^{s}n_{i}-\frac{\sum_{i}n_{i}}{\sum_{i}n_{i}}$$

In the above formulae, *n_i_* and *p_Ai_* denote the sample size and the frequency of SNP allele A in the *i*th population, respectively, and ${\bar{p}}_{A}$ is a weighted average of *p_A_* across populations. The negative Fst didn’t have any biological interpretation and were set to 0 to fit the definition of Fst ranging from between 0 and 1 (Wright, 1951). The top 1% of loci according to genetic differentiation values was served as candidate regions to host resistance or susceptibility to pig scrotal hernia (Zhang et al., 2019).

## Linkage Disequilibrium and Linkage Analysis (LDLA)

The haplotypes of F3 on SSC8 were reconstructed using a hidden Markov model by beagle (Zhang et al., 2012) and then the graphical model for the haplotype clusters with beagle was directly generated, which is a directed acyclic graph (DAG). The parameters for both processes are set to scale equals 2 and shift equals 0.1. Haplotypes within a cluster are likely to descend from the same ancestral haplotype and to carry the same DSV (DNA sequence variants) and combination of alleles, which is actually the principle used in linkage analysis. The linkage disequilibrium or association mapping information is generated by ancestral recombinations and detected by population level associations between individuals. Then, the clustered haplotypes were converted into diallelic markers by pseudomarker program, which can be imported into a program like R for statistical analysis. Thus, haplotype data contains both linkage and linkage disequilibrium information and can be imported into a mixed model framework:

Y=Xb+Zu+e

where Y is the vector of phenotypes, and b is fixed effects including sex and batch. The haplotypes could be treated as random here, as there are likely to be many of them, and some haplotypes will occur only a small number of times. Therefore, the random additive genetic effect following the distribution $u\sim N(0,\sigma_{u}^{2}G)$ , in which G is the individual–individual similarity matrix, and $\sigma_{u}^{2}$ is the polygenetic additive variance, and X and Z are incidence matrices for b and u, respectively. The residual random effect “e” following the distribution $e\sim N(0,\sigma_{e}^{2}I)$. The LDLA analysis was carried out using a homemade R scripts (**Supplementary Data Sheet 2**). The most likely position of the QTL was obtained by the 2-LOD drop method (Karim et al., 2011).

## Haplotype Sharing Analysis

The haplotypes in the target QTL region were constructed by fastPHASE. Firstly, we tried to find the sharing susceptibility haplotype by thoroughly scanning the haplotypes of affected individuals in F_3_ population. Then, we tried to identify whether the same sharing susceptibility haplotype existed in F_2_ affected individuals and tried to trace it to the F_1_ and F_0_ generations. It should be noted that we take the intersection of SNPs of F_3_ and F_2_ due to the different density of 50 and 60k chip.

## Conditional Association Test

To elucidate whether there are additional QTLs for scrotal hernia in the identified QTL region, we extracted genotypes of the top SNP and included as a covariate to the univariate linear mixed model, which was performed in the single-marker GWAS as we described above then performed a conditional test to retest the association between SNPs and phenotypes. If additional signal was detected, then there were multiple QTLs that cooperated to control scrotal hernia. Otherwise, there was only one QTL that affected scrotal hernia.

## Bootstrap Test

The bootstrap method is a resampling technique used to estimate statistics on a population by sampling a dataset with replacement (Efron and Tibshirani, 1993). It can be used to estimate summary statistics such as the mean, standard deviation, confidence interval, or correlation coefficient, which is done by repeatedly taking small samples, calculating the statistic, and taking the average of the calculated statistics. We herein carried out bootstrap test to verify the reliability of GWAS in this study. First, we randomly resampled for 1,000 times with replacement, in which some affected individuals can be sampled for multiple times, while some may be sampled for 0 times, the total number of affected individuals that may either increase or decrease, and the same resample results were acquired in unaffected individuals. Then, we conducted GWAS for 1,000 times to see if there were still significant signals in the susceptibility region which was identified in our study.

## Results

### Phenotype Statistics and SNP Characteristics After Quality Control

Incidences of scrotal hernia were estimated to be 0.7 and 2.7% in the ordinary F_3_ population and in the specially designed F_3_ population, respectively. It is obvious that the incidence of scrotal hernia in the specially designed F_3_ population was significantly higher than in the ordinary F_3_ population. Heritability for scrotal hernia was estimated at 0.39 using the standard linear mixed model, which implies that there is a genetic contribution to scrotal hernia.

After quality control, a total of 42,365 SNPs and 246 pigs had retained for further analyses. Imputation was produced using Beagle software. The summarization of imputation results is presented in **Table 2**. After imputation, a total of 46,483,626 SNPs for 246 individuals were obtained, and 18,756,672 SNPs were retained after filtering with MAF > 0.03. The average genotypic concordance rate was 84.8%, and the average correlation between best-guess and true variants reached with an average of 71% after we delete sites where R^2^ is equal to 0 and MAF is less than 0.03 (**Supplementary Figure S2**).

**Table 2:** The distribution of SNPs in different chromosomes.

Chr	Before QC	After QC
Chr1	4,735,710	1,871,922
Chr2	3,000,496	1,128,593
Chr3	2,841,406	1,161,477
Chr4	2,711,334	1,140,103
Chr5	2,259,813	971,685
Chr6	3,399,128	1,410,997
Chr7	2,647,087	1,094,716
Chr8	2,786,786	1,193,047
Chr9	2,942,680	1,159,718
Chr10	1,840,363	856,736
Chr11	1,894,832	815,114
Chr12	1,502,345	649,471
Chr13	3,648,208	1,351,511
Chr14	2,828,077	1,122,063
Chr15	2,743,392	1,095,268
Chr16	1,788,429	728,729
Chr17	1,544,662	562,261
Chr18	1,368,878	562,261
Whole genome	46,483,626	18,875,672

Chr, chromosome number; QC, quality control. The QC condition was MAF > 0.03.

## Summary of GWAS

**Figure 1:**
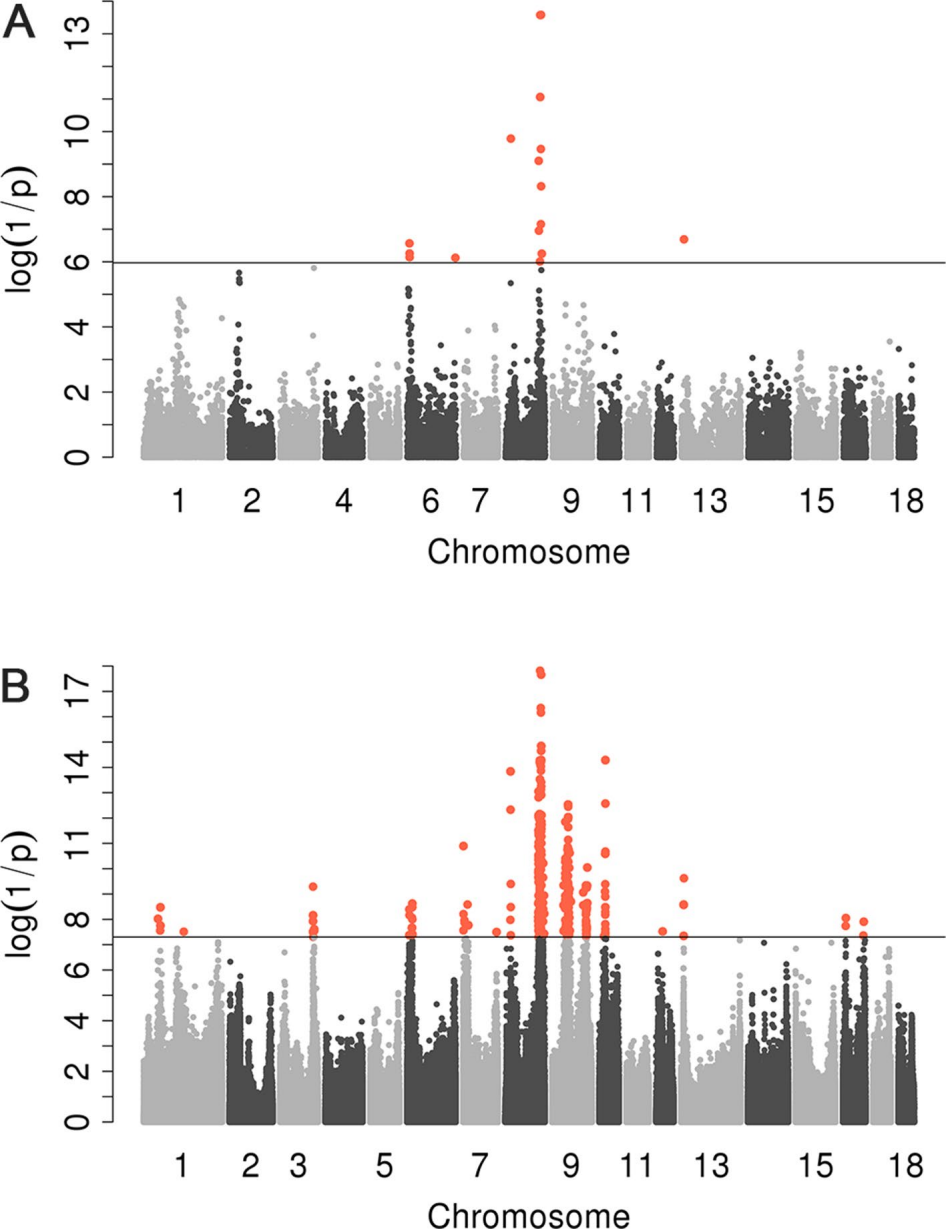
Manhattan plots for scrotal hernia with data before imputation and after imputation. log10 (1/P) are shown for all qualified SNPs, which were plotted against genomic position. In Manhattan plot **(A)**, black solid line indicates the 5% genome-wide significant threshold. In Manhattan **(B)**, the black line indicated the significance threshold [−log10(5E−08)]. All SNPs surpassing the genome-wide threshold are highlighted in pink.

We conducted a GWAS on the F_3_ population in two scenarios, i.e., the target data before and after imputations. In the scenario with experimental 50k chips data, we identified a total of 18 SNPs that surpassed the genome-wide significance level (**Figure 1A**). The most significantly associated SNP rs320409365 (*P-value* = 2.64 × e^−14^) locates at 124.1Mb within a 10.6Mb region (116.1–126.7Mb) on SSC8 (**Table 3**). In the scenario with imputed sequence data, 3,236 significant SNPs were located on SSC1, 3, 6, 7, 8, 9, 10, 12, 13, and 16 (**Figure 1B**), and the most significantly SNP rs319603861 (*P-value* = 1.52 × e^−18^) locates at 122.2Mb within a 21Mb region (115.5–136.5Mb) on SSC8 (**Table 4**). In addition, to validate the possibility of spurious SNPs caused by population stratification, the Q–Q plots for these GWAS were explored (**Supplementary Figure S3**). The average inflation factors (ကλ) of the GWAS were 1.17 and 1.2 in the two scenarios, respectively. Indicating that population structures were properly corrected.

**Table 3:** Description of the most significant 13 SNPs associated with scrotal hernia on chromosome 8 in F_3_ population with the 50k data.

Chr	ps	rs	P_wald
8	124,136,332	rs320409365	2.62E−14
8	121,414,739	rs333147082	2.64E−14
8	121,443,468	rs81404013	8.72E−12
8	121,025,652	rs318390967	8.72E−12
8	123,546,433	rs334430596	3.45E−10
8	116,106,612	rs329921419	7.94E−10
8	124,435,610	rs81306859	4.79E−09
8	123,575,503	rs327837715	7.01E−08
8	116,743,649	rs81284684	1.11E−07
8	126,744,562	rs81404481	5.65E−07
8	126,706,775	rs339470982	5.65E−07
8	120,387,726	rs81403944	9.81E−07
8	124,688,011	rs345674547	1.80E−06

Chr, chromosome number; rs, SNP IDs and SNPs that do not possess ID were named after Chr_ps, by the author; ps, base positions on the chromosome; P_wald, P-value from the Wald test.

**Table 4:** Description of the most significant 20 SNPs associated with scrotal hernia on chromosome 8 in F_3_ population with the data after imputation.

Chr	ps	rs	p_wald
8	122,211,833	rs319603861	1.53E−18
8	125,809,848	rs337122565	2.18E−18
8	125,809,964	rs339744702	2.18E−18
8	125,809,992	rs318592275	2.18E−18
8	124,541,337	rs695816095	4.51E−17
8	125,085,997	rs344335641	6.84E−17
8	125,845,805	rs321787225	1.42E−15
8	125,845,868	rs332303403	1.42E−15
8	126,802,357	8_126802357	1.44E−15
8	125,810,544	rs340831415	2.22E−15
8	125,818,781	rs324505118	2.22E−15
8	125,818,811	rs324505118	2.22E−15
8	125,810,459	rs327695191	5.07E−15
8	125,811,139	rs336507639	5.07E−15
8	125,812,250	rs81404378	5.07E−15
8	125,813,294	rs337489662	5.07E−15
8	125,817,665	rs321431992	5.07E−15
8	125,817,706	rs341020016	5.07E−15
8	120,993,480	rs790867883	5.29E−15

Chr, chromosome number; rs, SNP IDs and SNPs that do not possess ID were named after Chr_ps, by the author; ps, base positions on the chromosome; P_wald, P-value from the Wald test.

## Genetic Differentiation Scores

**Figure 2:**
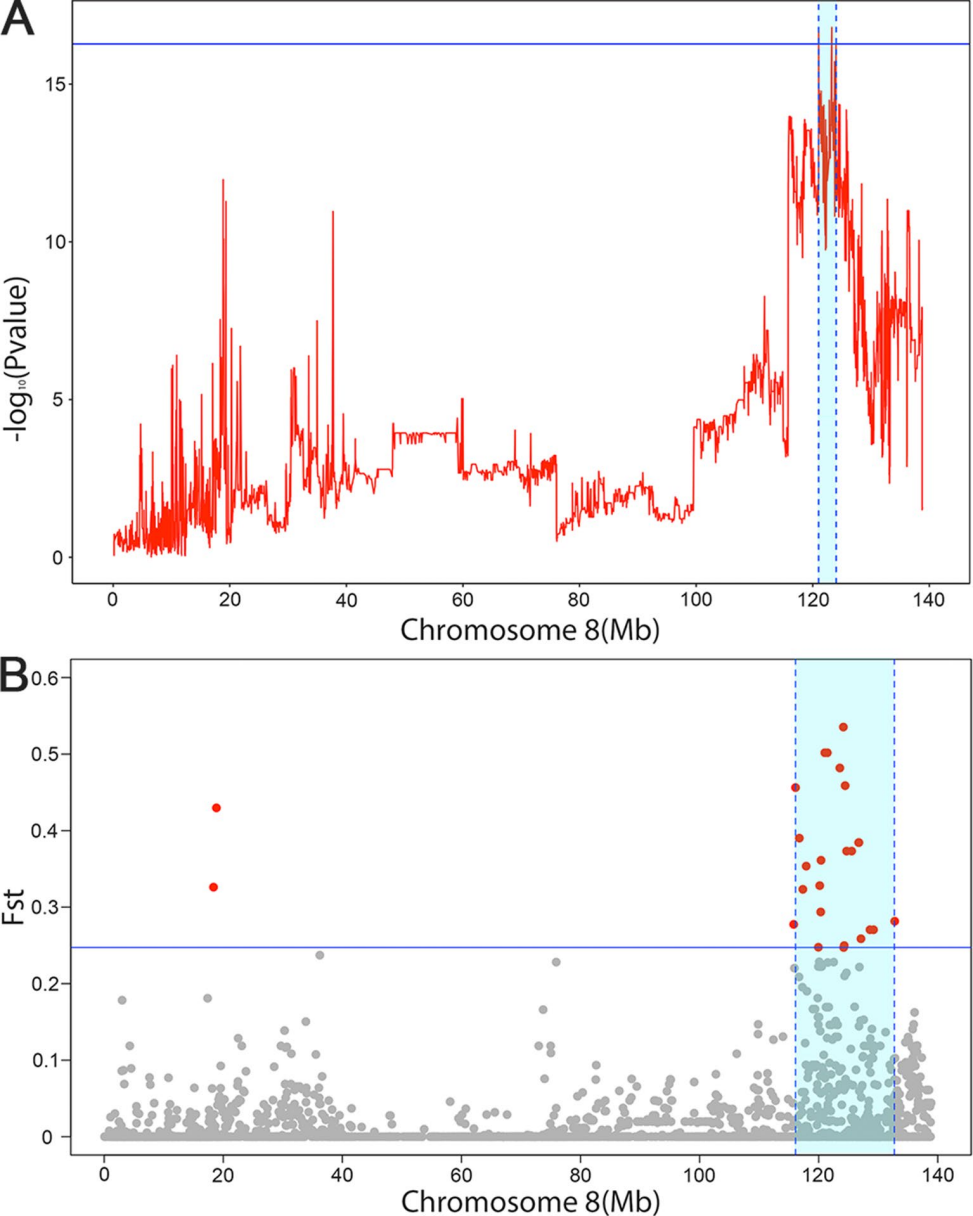
The significant associated region on SSC8 in LDLA analysis **(A)** and genetic differentiation analysis **(B)**. **(A)** The y-axis shows negative log_10_ (*P*-values) from haplotype-based association study, and the x-axis indicates the SNP positions on SSC8. The red lines represent the haplotype. The horizontal line indicated the 95% of confidence interval by LOD drop off two from the most significant haplotype. **(B)** The significant associated region on SSC8 were represented as light blue. The x-axis indicates the SNP positions on SSC8, and y-axis shows Fst. The horizontal line indicated the top 1 of confidence interval. All SNPs surpassing the threshold are highlighted in pink. Region with a large genetic differentiation were represented as light blue.

Fst were estimated to determine the extent of population differentiation between the affected and unaffected pigs. We identified a total of 26 SNPs beyond the empirical threshold on SSC8 (**Figure 2B**); the strongest genetic differentiation loci rs320409365 (Fst = 0.535) locates at 124.1Mb within a 16.6Mb region (116.1–132.7Mb), indicating the affected pigs and the unaffected pigs had a large genetic differentiation in this interval. All the SNPs beyond the empirical threshold in this interval are shown in **Table 5**.

**Table 5:** Genome-wide loci beyond the empirical threshold on chromosome 8 for pig inguinal/scrotal hernias identified by genetic differentiation analysis.

Chr	ps	rs	Fst
8	124,136,332	rs320409365	0.53536008
8	121,025,652	rs318390967	0.501778292
8	121,443,468	rs81404013	0.501778292
8	123,546,433	rs334430596	0.481862641
8	124,435,610	rs81306859	0.458791183
8	116,106,612	rs329921419	0.456265642
8	116,743,649	rs81284684	0.390162465
8	126,706,775	rs339470982	0.384398437
8	126,744,562	rs81404481	0.384398437
8	125,530,778	rs334269805	0.37324505
8	124,688,011	rs345674547	0.37324505
8	120,387,726	rs81403944	0.361238378
8	117,897,490	rs336417589	0.353602403
8	120,167,202	rs81403910	0.328123759
8	117,335,274	rs81324515	0.323287852
8	120,335,533	rs81403964	0.293693666
8	132,760,090	rs81323639	0.281592392
8	115,799,722	rs81307505	0.277478219
8	129,198,702	rs329385027	0.270487326
8	128,613,004	rs336466493	0.270487326
8	127,090,631	rs81317149	0.258692328
8	124,278,247	rs332687320	0.249736644
8	119,943,759	rs81330386	0.247590093
8	124,189,324	rs81340120	0.247278921

Chr, chromosome number; rs, SNP IDs and SNPs that do not possess ID were named after Chr_ps, by the author; ps, base positions on the chromosome; Fst, the genetic differentiation scores.

## Fine Mapping on SSC8 Using LDLA and Genetic Differentiation Analyses in the F_3_ Population

To further narrow down the confidence interval of SNPs SSC8 for scrotal hernia, we perform linkage and linkage disequilibrium (LDLA) for scrotal hernia on SSC8. The LDLA results showed the strongest association SNP was rs330263452 (*P*-value = 1.58 × 10^−17^); the most likely confidence interval of the QTL was approximately 3Mb (121–123.99Mb), based on the LOD drop off 2 (**Figure 2A**). We herein concluded a common QTL region located on SSC8 between 121.02 and 123.99Mb mapped by LDLA and genetic differentiation analysis.

## Haplotype Sharing Analysis Within the Confidence Interval

**Figure 3:**
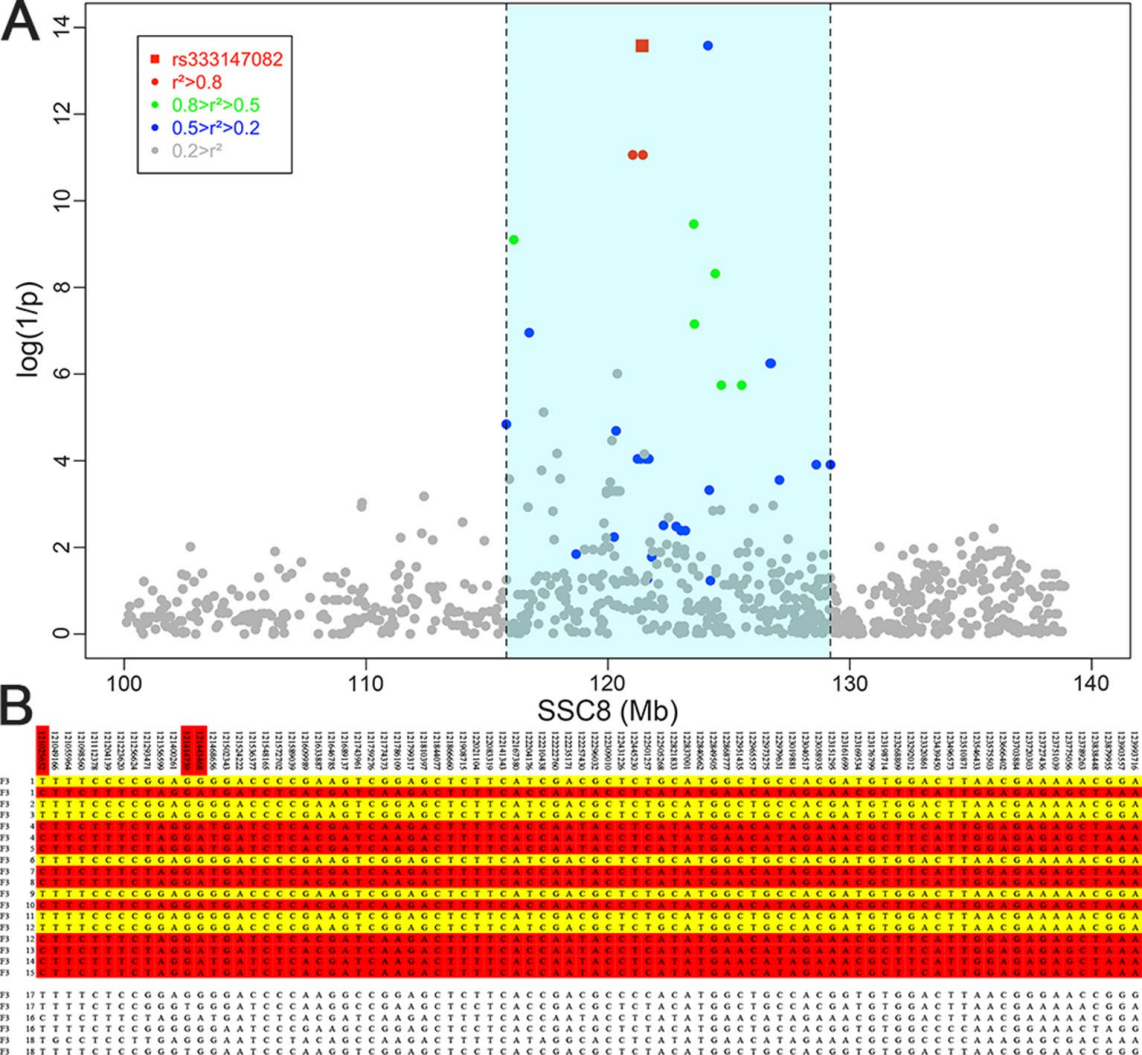
Fine mapping of the target region by the haplotype sharing analysis in the F_3_ population. **(A)** Regional association plot of SNPs in linkage disequilibrium with rs333147082. The colored diamonds indicate different linkage disequilibrium (LD) levels between rs333147082 and other SNPs. The light blue region indicates the interval which SNPs and rs333147082 with LD greater than 0.2. **(B)** Haplotypes of the target region between 121 ∼ 123.99 Mb on chromosome 8 are shown. Golden diamonds and red diamonds represent the Q_1_ and Q_2_ haplotypes with affected pigs, respectively. The last six lines indicate that three affected pigs who carried other types of haplotypes.

**Figure 4:**
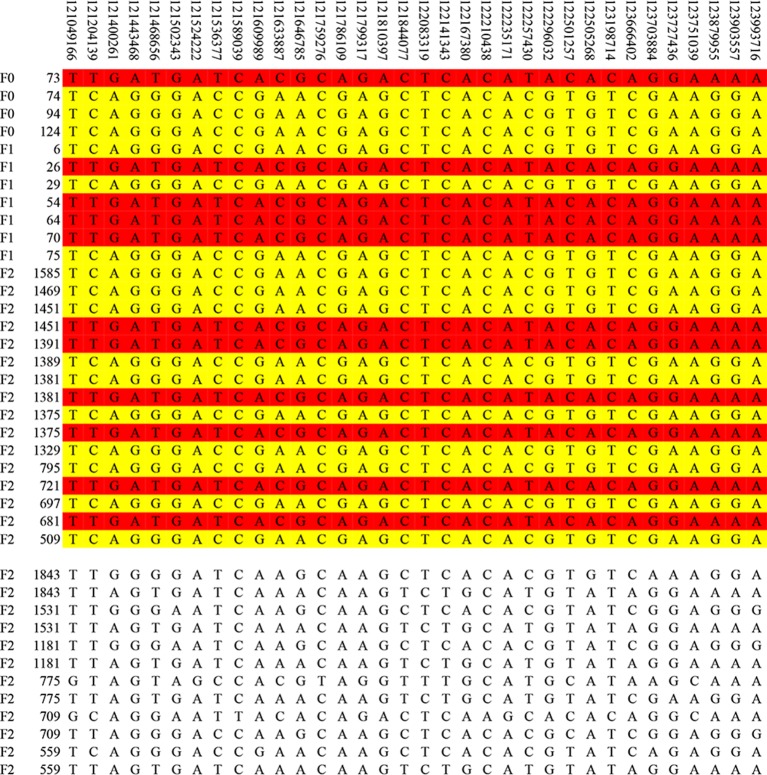
The haplotype sharing analysis in the F_2_ population. The figure showed that Q_1_ and Q_2_ haplotypes contained in F_2_ affected individuals and traced back to the F_1_ and F_0_ generations. The last 12 lines indicate six affected pigs that carried other types of haplotypes.

The result of haplotype sharing analysis on F_3_ population was showed on **Figure 3B**. To put the result in detail, 15 of 18th affected pigs shared two types of haplotype in this 2.97Mb region flanked by markers rs318390967 and rs81404172. Those two shared haplotypes were associated with pig scrotal hernia and presumably Q_1_-bearing and Q_2_-bearing haplotypes, respectively. Further investigation revealed 27 of 228 unaffected pigs also carried the Q_1_ or Q_2_ haplotype. To test the risk ration and significance of individual carried Q haplotype, we summarized the number of affected pigs and unaffected pigs who carried and uncarried Q haplotype and conducted chi-square test with them (chi-square test *P*-value = 8.46 × E^−15^). This result is indicative of that the hypothesized Q haplotype was involved in the occurrence of scrotal hernia in pigs. Next, we tried to identified whether there is the same sharing susceptibility haplotype existed in F_2_ affected individuals and trace this susceptibility haplotype back to the F_1_ and F_0_ generations. The result showed that 13 of the 19 affected pigs in the F_2_ population also carried Q_1_ or Q_2_ haplotype flanked by markers rs81275702 and rs81404172 (**Figure 4**), and another carried other types of haplotypes. According to the pedigree (**Table 6**), we also found that the parents of those 13 affected pigs also carried Q_1_ or Q_2_ haplotype in the same region, while the other 5 parents with other types of haplotypes individuals did not. Most of all, we found Q_1_ and Q_2_ haplotypes were come from of one White Duroc boars (F_0_-73) and three Chinese Erhualian sows (F_0_-74, F_0_-94, F_0_-124) when we traced those two haplotypes to the F_0_ generation, respectively. Therefore, it is concluded that two susceptibility haplotypes underlying the SSC8 were identified for pig scrotal hernia, and there should be some important pathogenic mutations. In addition, it was worth mentioning that the significantly associated SNP rs81404013 (*P*-value = 8.72×E^−12^), rs318390967 (*P*-value = 8.72×E^−12^), and rs333147082 (*P*-value = 2.64×E^−14^) that contained in this confidence interval have strong linkage disequilibrium extents (r^2^ > 0.9) to each other (**Figure 3A**). However, the most significantly associated SNP rs320409365 (*P*-value = 2.62×E^−14^) has a low linkage disequilibrium extents (r^2^ < 0.5) with those three loci. We take a region flanked by markers rs341392224 and rs326688253, which contain rs320409365, as well as it’s left and right two loci. Then, we count the types of haplotype in this interval and take a chi-square test with them (**Supplementary Table 1**); the result showed that haplotype CACGT (*P*-value = 1.02×E^−12^) was significantly associated with scrotal hernia.

**Table 6:** The pedigree of F_2_ affected individuals.

	Parent’s generation		Grandparent’s generation			
	Male	Female	Male	Female	Male	Female
F_2_-1,585	F_1_-29	F_1_-46	F_0_-75	F_0_-74	F_0_-73	F_0_-58
F_2_-1,469	F_1_-29	F_1_-52	F_0_-75	F_0_-74	F_0_-73	F_0_-58
F_2_-1,451	F_1_-75	F_1_-26	F_0_-75	F_0_-94	F_0_-73	F_0_-90
F_2_-1,391	F_1_-29	F_1_-64	F_0_-75	F_0_-74	F_0_-73	F_0_-202
F_2_-1,389	F_1_-29	F_1_-64	F_0_-75	F_0_-74	F_0_-73	F_0_-202
F_2_-1,381	F_1_-29	F_1_-64	F_0_-75	F_0_-74	F_0_-73	F_0_-202
F_2_-1,375	F_1_-29	F_1_-64	F_0_-75	F_0_-74	F_0_-73	F_0_-58
F_2_-1,329	F_1_-35	F_1_-6	F_0_-73	F_0_-58	F_0_-73	F_0_-124
F_2_-795	F_1_-75	F_1_-32	F_0_-75	F_0_-94	F_0_-73	F_0_-90
F_2_-721	F_1_-49	F_1_-70	F_0_-75	F_0_-94	F_0_-73	F_0_-202
F_2_-697	F_1_-29	F_1_-46	F_0_-75	F_0_-74	F_0_-73	F_0_-58
F_2_-681	F_1_-49	F_1_-54	F_0_-75	F_0_-94	F_0_-73	F_0_-58
F_2_-559	F_1_-3	F_1_-36	F_0_-73	F_0_-124	F_0_-75	F_0_-74
F_2_-509	F_1_-35	F_1_-6	F_0_-73	F_0_-58	F_0_-73	F_0_-124

## Candidate Gene *EIF4E* for Genome-Wide Significant QTL

The 2.95Mb region on SSC 8 in pig (Ensembl 2018) encompasses eight annotated genes (*ADH6, ADH4, ADH5, METAP1, EIF4E, TSPAN5, RAP1GDS1, STPG2*), which indicated that few genes are the most likely candidate genes that caused scrotal hernia in pigs (Zerbino et al., 2018). Of the eight genes, *EIF4E* stood out as a potential candidate based on its biochemical and physiological functions. *EIF4E* is a protein-coding gene, which regulates the expression of the Eukaryotic translation initiation factor 4E protein, and translation initiation factor 4E is regulating the expression of *MID1* gene (Pelletier et al., 1991; Jones et al., 1997). Winter et al. demonstrated that loss-of-function mutations in the *MID1* gene may cause the malformations of the ventral midline, which always lead to a series of urogenital abnormalities, such as cryptorchidism, ambiguous genitalia, hypoplastic scrotum, and umbilical and inguinal hernias (Winter et al., 2016). In addition, both top SNPs rs333147082 (*P*-value = 2.64×e^−14^) and rs81404013 (*P*-value = 8.72×e^−12^) located in the intron of *EIF4E* gene when we condition the strongest significantly associated SNP rs333147082; no additional association signals appeared in this loci (QTL) was detected (**Supplementary Figure S4**), which showed the additional evidence for the causality of *EIF4E* incorporating functional and conditional association studies. These results were more evidence that the *EIF4E* is the susceptibility gene for pig scrotal hernias.

## Discussion

In the current study, we obtained 18,756,672 variants with 84.8% genotypic concordance rate. In the study on imputation, few researches reported the imputation accuracy from 60K to whole-genome sequence in pig, compared to most studies focused on imputation from low-density genotypes to 60k variants with correlations ranging from 0.938 to 0.992 for imputation from 3 to 60K (Cleveland and Hickey, 2013). Yan et al. showed an average genotypic concordance of 89% with imputing 60K to whole-genome sequence variants in a large-scale swine F2 resource population (Yan et al., 2018), and Zhang et at. reported the genotypic concordance was 85.6% from 650K to whole-genome sequence variants using a stepwise imputation strategy in 1,363 Duroc pigs (Zhang et al., 2018); the genotypic concordance rate (84.8%) in our study is almost to their level. Moreover, we adopted R^2^ to estimate imputation accuracy on account of genotypic concordance rate that is highly sensitive to MAF and is not appropriate for comparing genotypes with different MAF (Yan et al., 2018).

In the present study, R^2^ decreased from 58 to 8% when MAF decreased from 0.1 to 0. The same trend was found in other studies (Daetwyler et al., 2014; Yan et al., 2018). And the average correlation between best-guess and true variants reached with an average of 71% after we delete sites where R^2^ is equal to 0 and MAF is less than 0.03. Besides, Yan et al. showed that the average correlation is lower than the genotypic concordance rate, which was consistent with our result in this study (Yan et al., 2017). In addition, there are many other factors that affect the accuracy of imputation, such as the relationships between target panel and reference (van Binsbergen et al., 2014) and LD and reference size (van Binsbergen et al., 2014). Here we sequenced 19 ancestors of F3 to ensure our imputation reliability. Overall, imputation accuracy can be affected by different aspects, and high accuracy of imputation will lead to a reliable GWAS.

GWAS has become an exceedingly effective and widely used approach in identification of genetic variants associated with common diseases or complex traits since the first application of GWAS research was performed successfully in 2005 by Klein et al. (2005). Previously, by performing haplotype-based GWAS in F_2_ population for scrotal hernia using Porcine SNP60 BeadChip, 108 chromosome-wise significance SNPs were identified to be associated with scrotal hernia; however, there was no marker surpassed the genome-wide significance level. The feasible reasons for the low detection power in this study was probably the low incidence and penetrance rate in F_2_ population. But the most possible reason is the intricacy molecular genetic mechanism of scrotal hernia. So far, many international teams have identified the susceptibility loci of scrotal hernia on almost all chromosomes. Complex interactions between environmental factors and susceptibility alleles of multiple genes are the most normal process resulting in such a complex genetic background diseases. As a complex genetic defect, the polygene model may be the main pathogenesis, under polygene model that lots of susceptibility genes cause a change for disease. Therefore, whether a certain mutation is not directly related to scrotal hernia, but does have a role in the occurrence of it, this is why single-marker GWAS can’t detect any significant signal in the F_2_ population. Therefore, we generate a particular hernia population which was mated with full-sibs or half-sib of the affected individuals. The incidences of scrotal hernia will increase significantly in this population. Next, we will systematically describe the feasibility of our idea.

In the current study, we first designed a specially F_3_ population to increase the incidence and penetrance rate by crossing full-sibs or half-sib of the affected individuals in the F_2_ population. Statistics manifested that prevalence of scrotal hernia in the specially designed F_3_ population was 3.6 times and 2.4 times higher than the ordinary F_3_ population and F_2_ population, respectively, indicating the F_3_ specially designed population is completely successful in increasing the incidence rate of scrotal hernia. Most importantly, in the F_2_ population, the frequency of a mutation associated with scrotal hernia will be greatly increased in F_3_ specially designed population, as the health full-sibs or half-sib of the affected individuals in the F_2_ population also have the mutant sites, which will pass on to the F_3_ population.

As we predicted, 13 SNPs were located on SSC8 between 116.1 and 126.7Mb surpassed the genome-wide significance level after we conducted a GWAS on the specially designed F_3_ population with experimental 50-k chip data, and this QTL must have come from F_2_, which overlaps with a region previously identified by Sevillano et al. (2015). The basic principle of single-marker GWAS was to test association between phenotypes and genotypes. Normally, this association was indirect correlation as the causative mutation was not included in the study locus. Potentially, significant signals could be missed in a GWAS analysis if there were low LDs among paired markers. To improve the LD between markers, we performed imputation analysis by increasing the marker density in the study population using 109 sequenced data as reference panel. Consequently, we obtained 18,756,672 variants with relatively high imputation accuracy (average CR = 84.8%). After performing the whole-genome association study with sequence data, 3,252 significant SNPs reached the significant level. Three regions located on SSC3, SSC8, and SSC10 were similar to corresponding interval previously identified by Sevillano et al. (2015), especially the region on SSC8 between 115.6 and 136.5Mb overlaps a region they previously identified. To our knowledge, it is the first time that the other eight QTL regions identified on SSC1, 6, 7, 9, 13, and 16 are found to be associated with scrotal hernia, although some studies have reported that different regions on these chromosomes harbor QTL for scrotal hernia.

According to our original intention, we identified 13 SNP loci significantly associated with scrotal hernia on chromosome 8 through GWAS analysis with the specially designed F_3_ population. In the subsequent analysis, we divided the affected individuals and unaffected individuals into two independent groups and calculated the genetic differentiation index to verify that there is genetic differentiation on SSC8. The result showed that a strong genetic differentiation signal located on rs320409365 (Fst = 0.535) within a 16.6Mb region (116.1–132.7Mb) on SSC 8 was detected. This result indicated that the affected pigs and the unaffected pigs had a greater genetic differentiation in this confidence interval. Moreover, there is a high coincidence of the top SNPs detected through genetic differentiation analyses and GWAS.

Additionally, in consideration of single-marker GWAS, it was hard to properly estimate the confidence interval of the detected QTL, as LD varied severely among nearby SNPs while haplotypes have stable LD than SNPs. Thus, we conducted haplotype-based LDLA analysis, by simultaneously taking advantage of recent and ancestral recombination events to increase the efficiency and detect confidence interval. The LDLA results showed that the SNP with the strongest association at the locus was rs330263452 (*P*-value = 1.58 × 10^−17^), and the most likely confidence intervals around the 121–123.99Mb region on SSC8. Furthermore, we found out that the confidence intervals mapped by LDLA contained within the region mapped by genetic differentiation analysis. We narrow the confidence interval to 2.99Mb by picking up the intersection of those two intervals for further analysis.

Lastly, we identified two susceptibility haplotypes underlying the SSC8 associated with scrotal hernia after performed a haplotype sharing analysis, and those two haplotypes were from one White Duroc boar (F_0_-73) and three Chinese Erhualian sows (F_0_-74, F_0_-94, F_0_-124), respectively. It is incomprehensible that the White Duroc boar (F_0_-73) carried the susceptibility haplotype, but it was unaffected. Actually, whether a certain mutation is not directly related to scrotal hernia as we explained earlier. Similarly, 163 of 497 unaffected pigs in the F_2_ population also carried the susceptibility haplotypes, echoing the result that there was no significant signal when we performed GWAS in the F_2_ population. When we merge the F_2_ and F_3_ populations and then conducted chi-square test with them (chi-square test *P*-value = 8.32×E^−11^), this result is also indicative of that the hypothesized Q haplotype was involved in the occurrence of scrotal hernia in pigs. In addition to discovering two susceptibility haplotypes, we further found that there are nine annotated genes in this 2.95Mb interval in total, and the *EIF4E* was selected as potential candidate gene based on its biochemical and physiological functions.

Although there are some crucial discoveries revealed by these studies, there are a slice of limitations to our study, such as the relatively small number of samples in the F3 population. Therefore, we herein carried out bootstrap test to verify the reliability of GWAS in this study. The result showed that there are 957 of the 1,000 GWAS that were detected significant signals in the 116–126Mb interval on chromosome 8, which indicated that the fluctuation in the number of affected and unaffected individuals has no effect on GWAS (FDR < 0.05). Therefore, the significant signals obtained in our GWA study were not accidental but were caused by differences in the genomes of affected and unaffected individuals, which were reliable.

## Conclusion

In summary, in the first place, we discovered a major quantitative trait loci (QTL) for pig scrotal hernia on chromosome 8 in an F_3_ specially designed population using GWAS. There is one more point: two susceptibility haplotypes (Q_1_ and Q_2_) flanked by markers rs81275702 and rs81404172 and one potential causal gene underlying the SSC8 were identified through a series of methods including genetic differentiation analysis, LDLA, and haplotype sharing analysis. Last but not the least, we explain why many international research teams do not have a high repeatability of the results of scrotal hernia research, and some research studies haven’t even found any associated locus with scrotal hernia. Further studies will be devoted to confirming the detected haplotype and gene in outbred populations.

## Data Availability

The genotypic data of 246 F3 individuals as well as the phenotype of scrotal hernia for this study can be found in the figshare Digital Repository (https://figshare.com/s/d661962aa6c0740caeab), and the genotypic data of 516 F2 individuals analyzed for this study can be found in the Dryad Digital Repository (https://doi.org/10.5061/dryad.7kn7r) (Ma et al., 2013). The raw reads of the whole-genome sequence can be found from the NCBI sequence read archive (SRA) under the accession codes SRA065461 and SRP159212 (Ai et al., 2015; Yan et al., 2018).

## Ethics Statement

This study was approved by the ethics committee of Jiangxi Agricultural University. All procedures including experimental animals established and tissue collection were performed in accordance with the guidelines approved by the Ministry of Agriculture of China.

## Author Contributions

LH and ZZ conceived and designed the experiments. WX, GY, and TH analyzed the data. DC and SX contributed materials and analysis tools. WX, ZZ, and LH wrote the manuscript. All authors read and approved the final manuscript.

## Funding

This work was supported by National Natural Science Foundation of China (31760656) and Guangdong Sail Plan Introduction of Innovative and Entrepreneurship Research Team Program (No. 2016YT03H062).

## Conflict of Interest Statement

The authors declare that the research was conducted in the absence of any commercial or financial relationships that could be construed as a potential conflict of interest.
