# Supplementary material for: Rediscover and Refine QTLs for Pig Scrotal Hernia by Increasing a Specially Designed F3 Population and Using Whole-Genome Sequence Imputation Technology
Source: Front Genet. 2019 Sep 23;10:890. doi: 10.3389/fgene.2019.00890 (PMC6768097; doi:10.3389/fgene.2019.00890)
Supplement: Supplementary file 1 [file DataSheet_1.pdf]

SUPPLEMENTARY MATERIAL:

Title: Rediscover and Refine QTLs for Pig Scrotal Hernia by Increasing a Specially Designed F_3 Population and Using Whole Genome Sequence Imputation Technology

Wenwu Xu, Dong Chen, Guorong Yan, Shijun Xiao, Tao Huang, Zhiyan Zhang† and lusheng Huang†

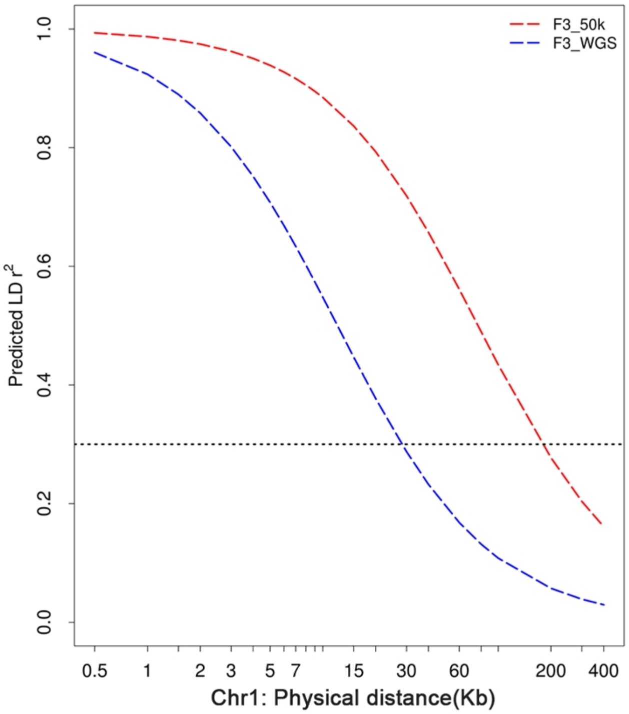

Figure S1 Linkage disequilibrium (LD) decay for F3 individuals in chromosome 1. LD decay was calculated for F3 using 50K genotype (*red line*) and whole-genome imputed data (*blue line*). X-axis indicates the physical distance (kb) between two markers, and if distances between two markers is greater than 400kb, the LD will not be calculated. Y-axis indicates the predicted LD by non-linear fit.

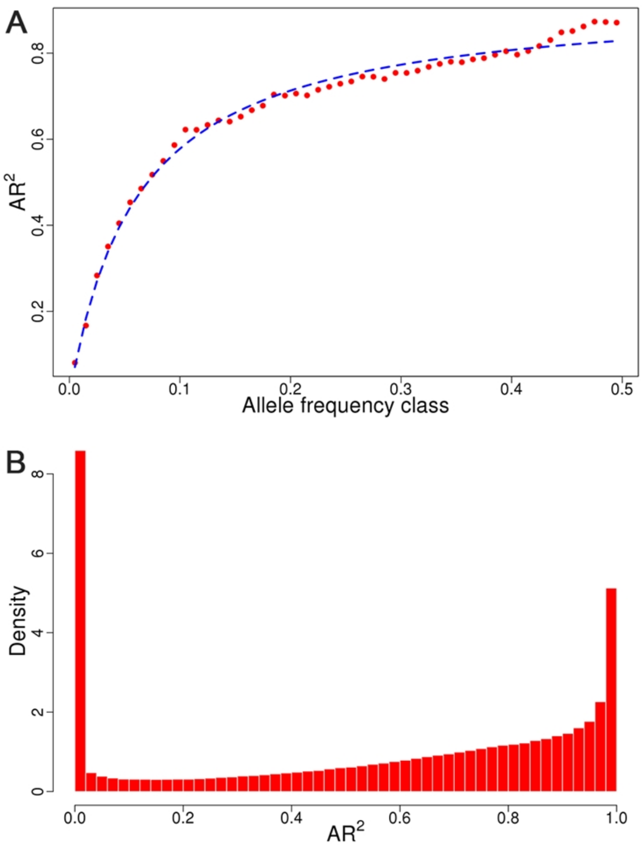

Figure S2 imputation accuracy. (A) The squared correlation between sequenced and imputed individuals, the x-axis is the MAF range from 0 to 0.5, and the y-axis is imputation accuracy denoted by the AR2. And the blue line indicates the predicted AR2 by non-linear fit. (B) the histogram of AR2.

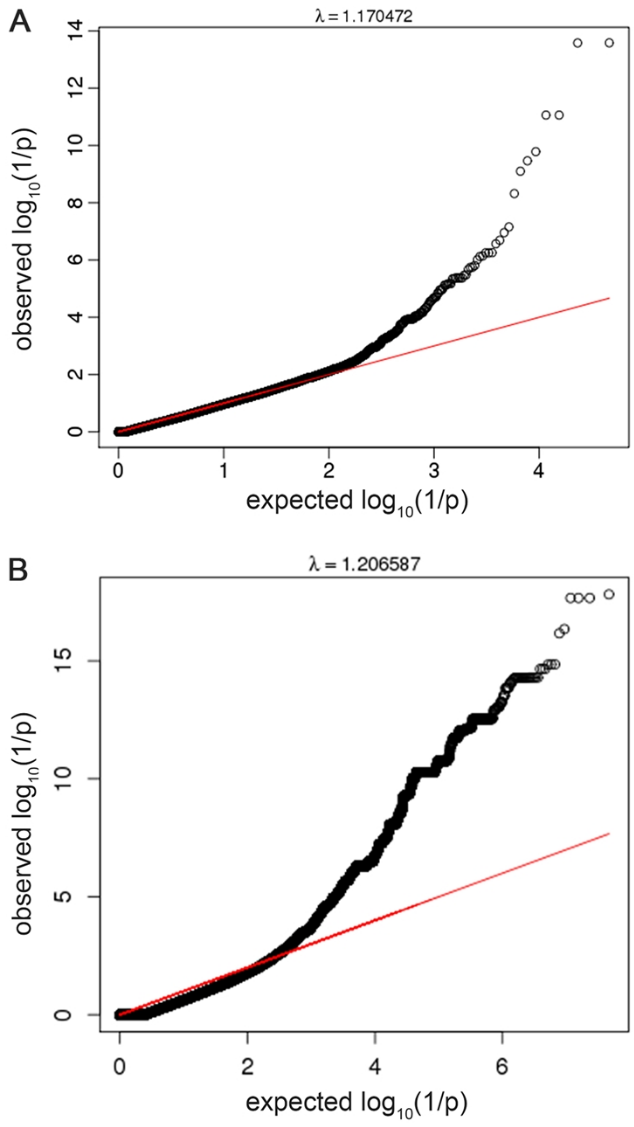

Figure S3 Quantile-quantile (Q-Q) plots for influence of population stratification on GWAS. The y-axis and x-axis represent the expected and observed negative $\log_{10}$ *P*-value, respectively.

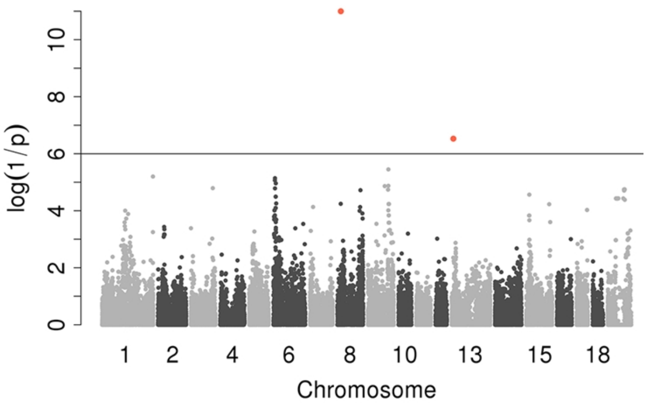

Figure S4 Manhattan plot of conditional GWAS for F_3 populations. Log(1/p) of SNPs were plotted against their genomic position, the solid line indicates the 5% genome-wide significant threshold. SNPs surpassing the genome-wide threshold are highlighted in pink.

Table S1 Haplotypes of the region between flanked by markers rs341392224 and rs326688253 on chromosome 8 are shown (quantity greater than 10), and the chi-square result of it.

**Haplotype**	**case**	**control**	***P*-value**
CACGT	12	17	1.02E-12
CATAC	4	76	0.35
CATAT	1	144	2E-4
CGTGC	2	31	0.74
TGCAC	11	35	9.5E-06
TGTAC	6	150	0.035
