# Supplementary material for: Rediscover and Refine QTLs for Pig Scrotal Hernia by Increasing a Specially Designed F3 Population and Using Whole-Genome Sequence Imputation Technology
Source: Front Genet. 2019 Sep 23;10:890. doi: 10.3389/fgene.2019.00890 (PMC6768097; doi:10.3389/fgene.2019.00890)
Supplement: Supplementary file 2 [file DataSheet_2.docx]

#This function was used to do GWAS with LDLA model. we need prepare phenotype data and Hidden states data

#the first column for phe and hidden states should be the recode id, which will be used to merge.

#the result was compare two model with and without haplotype effects with a F test.

#phe at least with id and hap at least with id

LALD <- function(formula,phe,hap) {

# the formula should be as string because formula :H1 <- lm(as.formula(paste(formula,' + Z',sep=''))) were use downline

if (typeof(formula)=="language") {

class(formula) <- 'character'

formula <- paste(formula[2],formula[1],formula[3])

}

numPar <- unlist(strsplit(formula,'~'))

hap <- as.data.frame(hap)

phe <- as.data.frame(phe)

#deal with NA's for phenotypes

colnames(phe)[1] <-'id'

colnames(hap)[1] <- 'id'

ncolPhe <- ncol(phe)

ncolhap <- ncol(hap)

hap <- merge(hap,phe,by='id')

hap <- hap[order(hap$id),]

phe <-hap[seq(1,nrow(hap),2),ncolhap:ncol(hap)]

hap <- hap[,3:ncolhap]

attach(phe)

#H0 <- lm(as.formula(formula),na.action='na.omit')

test_oneMK <- function(hapone){

if (max(hapone)==1) {

res <- c(0,0,0,0,1)

} else {

Z <- matrix(0,nrow=nrow(phe),ncol=max(hapone))

Z[cbind(1:nrow(phe),hapone[seq(1,length(hapone),2)])] <- Z[cbind(1:nrow(phe),hapone[seq(1,length(hapone),2)])] + 1

Z[cbind(1:nrow(phe),hapone[seq(2,length(hapone),2)])] <- Z[cbind(1:nrow(phe),hapone[seq(2,length(hapone),2)])] + 1

rm.col <- which(colSums(Z)<1)

if (length(rm.col)>0){

Z <- Z[,-rm.col]

}

if(length(numPar)>1){

H1 <- lm(as.formula(paste(formula,' + Z',sep='')),na.action='na.omit') #线性模型

} else {

H1 <- lm(as.formula(paste(numPar[1],' ~ Z',sep='')),na.action='na.omit')

}

res <-anova(H1)

res <- c(res$F[rownames(res)=='Z'],res$Df[rownames(res)=='Z'],res$Df[rownames(res)=='Residuals'],

res$Sum[rownames(res)=='Z']/sum(res$Sum),res$Pr[rownames(res)=='Z'])

res

}

}

myresult <- apply(hap,2,test_oneMK)

myresult <- t(myresult)

detach(phe)

colnames(myresult) <- c('Fvalue','Df1','Df2','var_pct','pval')

return(myresult)

}

########### test ######################################

phe <-cbind(1:200,rnorm(200,10,3))

hap <-matrix(sample(1:20,2000,replace=T),nrow=400)

id <- sort(rep(phe[,1],2))

hap <- cbind(id,hap)

colnames(phe) <- c('id','testphe')

test <- LALD(formula=testphe~1,phe,hap)
